# Depression in Patients With Intracranial Hemorrhage Secondary to Traumatic Brain Injury

**DOI:** 10.7759/cureus.42147

**Published:** 2023-07-19

**Authors:** Yasmine Rifai, Nicholas Cassimatis, Bruce Rubenstein M.D.

**Affiliations:** 1 General Surgery, Hackensack University Medical Center, Hackensack, USA; 2 Neurological Surgery, Hackensack University Medical Center, Hackensack, USA; 3 Psychiatry, Hackensack University Medical Center, Hackensack, USA

**Keywords:** antidepressants, emergency neurosurgery, alcohol use disorder, intracranial hemorrhage, depression, traumatic brain injury, tbi, selective serotonin reuptake inhibitor (ssri)

## Abstract

This article discusses the prevalence of depression in patients with intracranial hemorrhage (ICH) and the relationship of selective serotonin reuptake inhibitor (SSRI) use with bleeding risk. A detailed account of the patient’s psychiatric history and current hospital admission is also provided. This article then further explores the pathophysiological mechanisms that contribute to depression in ICH patients, the effect of SSRIs on outcomes in patients with ICH, and ways to treat depression in ICH patients. Based on the literature, the conclusion is that practitioners should avoid SSRIs in ICH patients with certain genetic markers and treat depression as seriously as one would treat a physical ailment. Ultimately, more research is necessary to explore how to treat depression in this patient population.

## Introduction

This case explores the different mechanisms by which depression and intracranial hemorrhage (ICH) may impact each other. Depressive symptoms in patients with intracranial bleeds are exceedingly common, with up to 20% of patients being diagnosed with depression [1]. Another study estimates that 25-50% of patients with traumatic brain injury (TBI) experience depression after the injury, with lifetime rates of 26-64% [2]. While the patient presented in this report had a prior diagnosis of major depressive disorder (MDD), it is also likely that the underlying pathophysiological mechanisms involved in the ICH may have exacerbated her MDD, contributing to her suicidal ideation at the time of admission. Common symptoms seen in relation to depression secondary to TBI include memory impairments, sleep disturbances, headache, and mood disorders [3]. This patient experienced frequent lapses in memory, had issues with sleep, experienced headaches, and had suicidal ideations.

## Case presentation

The patient is a 42-year-old female with a past psychiatric history of alcohol use disorder, MDD, a past medical history of ICH, and new onset seizure; she was being evaluated by psychiatry for suicidal ideation. This patient’s case is complex as there are multiple components that factor into her presentation on this admission, including suffering from an intracranial bleed after a TBI, extensive history of alcohol use disorder, and a history of MDD.

Psychiatric history

The patient's sister was a major source of history in this case as the patient was frequently lethargic and unable to adequately communicate with the treatment team. The sister states that the patient has been dealing with depression for at least 10 years. The patient has seen four psychiatrists in the past for depression and fatigue. During her last visit with her psychiatrist 5 weeks ago, she was prescribed topiramate, trazodone, and escitalopram. The sister stated that the patient endorsed feeling better on the medication, but then she missed a few doses and started drinking again. The patient has also had multiple episodes of suicidal ideation and attempts. The patient told the sister repeatedly that she drinks heavily because she wants to end her life. Nine weeks ago, the patient called her sister threatening to overdose on medication but did not go through with it. On the day of this current admission, the patient tried to end her life again with a medication overdose. Also, the patient’s father killed himself a few years ago, and her ex-wife attempted suicide a few months ago. Sister states these events were very distressing to the patient.

Additionally, the sister stated that the patient has been drinking heavily for at least 15 years and drinks hard liquor every day. The patient’s drinking led to her loss of employment, divorce with her wife, and multiple ER visits due to being found unconscious in her yard or in public areas. The patient has had multiple cases of driving while intoxicated and had been arrested. The sister notes that the family has constantly had to financially support the patient and that she has taken a lot of their money. She has been placed in rehab for alcohol use disorder on four separate occasions.

When interviewing the patient, she presented with confusion, lethargy, and slow processing. She endorsed wanting to throw herself off the top floor of the hospital, but she denied having a history of suicide attempts. When asked what brought her into the hospital, she stated she is in the hospital due to her drinking problem. During questioning, the patient confuses this admission with her previous admission. She also believes she's only been in the hospital for 24 hours, though it was day 8 of her admission when the team was interviewing her. She attempted calling her sister and brother several times, begging them to take her home. She tried walking out of the hospital multiple times and had to be brought back to her room by security. The patient asked where her ex-wife was multiple times and seemed to have forgotten that they were divorced. The patient endorsed symptoms of depression such as helplessness, hopelessness, changes in sleep patterns, and suicidal ideation. She denied anhedonia, decreased interest in previously enjoyable activities, feelings of guilt, lack of energy, trouble concentrating, changes in appetite, psychomotor slowing, or irritability. The patient denied auditory or visual hallucinations, as well as paranoid ideations, and did not appear overtly internally preoccupied.

Hospital admissions

**Figure 1:**
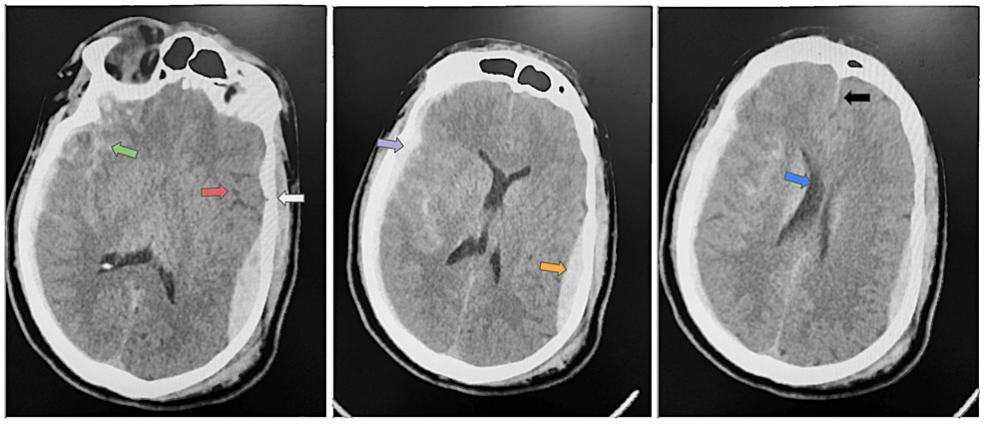
First series of head CT scans after the initial fall. Radiology report reveals a 5-mm shift of septum pellucidum from the right to left (blue arrow), areas of hemorrhagic contusion shear injury in the left temporal lobe (red arrow), and fracture at the left temporal squamosa (white arrow). Epidural hemorrhage can be seen in the left temporal region (orange arrow). Contrecoup injury can be seen in the right temporal region (green arrow). Acute subdural bleed can be seen along the right side of the temporal region (purple arrow).

In the patient’s last admission five weeks prior to this current admission, she was brought in due to a fall. She was found on the front porch of her home and was intoxicated due to alcohol use. The patient had multiple facial fractures, left temporal bone fracture, and right orbital fracture. She also had an epidural hematoma, subdural hematoma, and a contrecoup injury (Figure 1). After the patient was stabilized and had undergone treatment by the neurosurgery and trauma teams, she was discharged and sent home.

On the day of this current admission, 13 days after discharge, the patient was found by her sister suffering from a seizure on the kitchen floor. The patient presented with altered mental status and repeated statements consisting of suicidal ideation.

**Figure 2:**
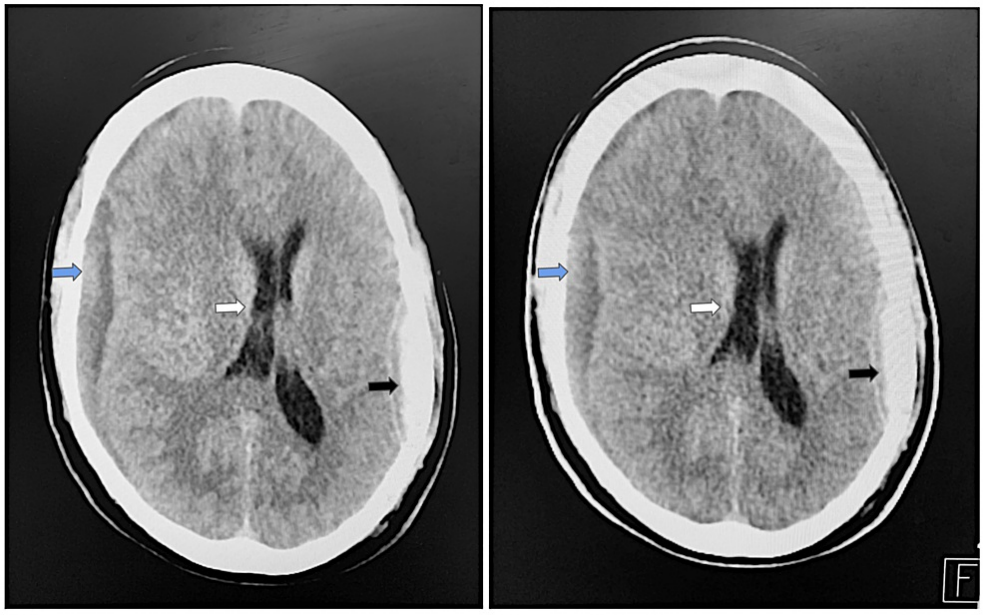
Day 1 of the second admission: second series of head CT scans after seizure. Radiology report revealed a stable subdural bleed (blue arrows) with right to left midline shift, stable epidural hematoma projecting over the left hemisphere (black arrows), and slightly increased local mass effect of the right frontal and temporal lobes with displacement of midline structures to the left with subfalcine herniation (white arrows).

On the way to the hospital, the patient was in a postictal state and was given ativan. During the hospital stay, an extensive workup was conducted. Her repeat CT scans (Figure 2) were stable compared to her last admission. However, due to the patient’s significantly altered mental status, she was admitted to the surgical intensive care unit for repeat monitoring with serial CT scans and neurological checks. Due to the patient’s continued expression of suicide ideation, she had a one-to-one sitter.

Furthermore, the patient’s medication regimen had to be adjusted. Escitalopram and trazodone were discontinued, and topiramate, depakote, and keppra were started.

On day 7, the patient had undergone right-sided burr hole evacuation to relieve her subdural bleed. The final CT scan showed a decrease in the size of the subdural bleed. After the procedure, the patient was persistently denying suicidal ideation for one week. She was then cleared for discharge by psychiatry. The psychiatry team had not made any further changes to her medication regimen prior to discharge.

Differential diagnoses

While the patient had a previous diagnosis of MDD, there are other possible diagnoses that could contribute to this patient’s presentation. These include a substance-induced mood disorder and a TBI/ICH-induced mood disorder. Alcohol is an indirect GABA (gamma-aminobutyric acid) agonist and leads to the elevation of serotonin and dopamine. Without alcohol, a patient becomes deficient in these neurotransmitters due to negative feedback mechanisms, which can lead to a depressed mood. The discussion below will consist of how TBI and ICH can exacerbate or cause depression.

## Discussion

Depression in patients with intracranial bleeds and TBI

While this patient has a previous diagnosis of MDD, it is also important to consider the role that her physical illness may play in exacerbating her symptoms.

One study examines multiple complex pathophysiological mechanisms that play into the presentation of depression in patients with ICH (intracerebral hemorrhage) [3]. These factors are commonly related to secondary brain injuries from the bleed, which lead to various changes through inflammatory, oxidative, and apoptotic pathways [3]. Inflammation causes white blood cells and red blood cells to travel to the brain parenchyma, which causes impairment of brain function [3]. Oxidative stress pathways are triggered secondary to ICHs, and one of those pathways involves Nrf2 [3]. A separate study identified that depression is caused by the distortion of six different pathways, one of which involves Nrf2 [3]. Finally, ICH leads to activation of caspases, causing apoptosis [3]. In the pathophysiology of depression, apoptosis plays a role by destroying serotonergic neurons [3]. Evidently, there are a multitude of intricate mechanisms that link the pathophysiology of ICH with depression [3].

It is also important to note that this patient’s ICH was secondary to a TBI, which was caused by the patient’s fall. Another study [4] examines the psychiatric consequences patients experience after a TBI. Depression was identified as a common long-term complication following a TBI. Again, this was found to be related to chronic inflammatory processes, such as marked increase in cytokine levels such as tumor necrosis factor-a and interleukin-1 [4]. These cytokines can cause changes by directly changing neuronal synapse physiology [4]. The study showed that these inflammatory cytokines remain above normal, physiological levels for months to years after the injury, which can subsequently lead to persistence of depressive symptoms for months to years [4].

Another interesting facet is that depression itself may worsen outcomes in patients with intracranial bleeds. A study of the DASH (diagnostic accuracy of MRI in intracerebral hemorrhage) study included 89 subjects that were examined for depression one year after experiencing hemorrhage [5]. Results showed that depressed patients had worse one-year outcomes (p=0.004) [5]. However, it can be difficult to discern whether these patients did worse because they were depressed or if they were depressed because they were suffering from more physical-related morbidities than their non-depressed counterparts [5].

Antidepressant use in intracranial bleeds

An important issue that came up during the management of this patient was the use of antidepressants. The patient’s sister insisted that escitalopram was very helpful for her mood, and the patient endorsed this sentiment as well. However, the treatment team decided against using selective serotonin reuptake inhibitors (SSRIs) due to the patient’s ICH. This is because literature has shown that serotonin reuptake causes platelet dysfunction.

The ERICH (Ethnic/Racial Variations of Intracerebral Hemorrhage) study examined the effects of SSRIs on bleeding complications in patients with ICHs [6]. It examined a large, prospective case-control study that recruited 1,000 non-Hispanic white, 1,000 non-Hispanic black, and 1,000 Hispanic patients with ICH with matched ICH-free controls [6]. Participants were enrolled from 19 U.S. sites and 42 hospitals [6]. The results of the study found that post-ICH SSRI use was associated with an unfavorable 3-month neurological outcome after ICH [6]. Neurological outcomes were defined as mRS (modified Rankin scale) ≥3 [6]. While neurological outcomes were adversely affected, there were no observed changes on repeat head CT of these patients [6]. Considering this finding, it could be argued that the worsened neurological outcomes may be related to depression itself, rather than a sequelae of the physical illness. However, more studies with more in-depth protocols would be needed to support this hypothesis.

Additionally, Fann et al. examined 12-week treatment responses to sertraline in 62 randomized participants with a TBI [7]. Many of these patients had a previous history of depression or depression post-TBI [7]. The study found that after 12 weeks of sertraline and placebo, the sertraline group did not experience statistically significant improvements in depressive symptoms compared to the placebo group [7].

Treating depression in post-ICH patients

The evidence shows that not only antidepressants may worsen physical outcomes in patients with ICH but they may also not help treat depressive symptoms. After explaining this to the patient’s family, the next question was regarding how to manage the patient’s depressive symptoms with this concurrent comorbidity. After the burr hole evacuation surgery, the psychiatry team did not make any changes to the patient’s medical regimen. The literature also showed that there is a lack of national or professional guidelines regarding the treatment of depression in post-ICH patients.

One study examined the effects of fluoxetine in these patient populations. This SSRI was shown to improve depression in post-ischemic stroke patients [1]. However, this same finding has not been replicated in patients with depression after ICH [1]. There is currently no treatment for depression in post-ICH patients that is approved by the U.S. FDA [1].

A different study explores the use of genetic biomarkers to guide the risk of using SSRIs in post-ICH patients with depression [8]. It demonstrated that SSRI use for depression following ICH resulted in higher likelihood of remission of depression, but in certain individuals, it led to increased risk of repeated hemorrhage [8]. Some of the markers that they discovered included APOE e2/e4, as this marker is associated with cerebral amyloid angiopathy and thus increases the chance of ICH [8]. Thus, the conclusion of this study is that SSRIs may be used in post-ICH patients if they lack these genetic markers [8].

## Conclusions

Overall, it is apparent that more research is needed to guide treatment plans in depressed patients post-ICH. Using SSRIs with caution is necessary as SSRIs may lead to exacerbation of an intracranial bleed. Ultimately, the most important takeaway from this case is that treating comorbid depression in this patient population should be as vital as treating the bleed itself. Allowing depression to expand and consume a patient’s life can lead to severe physical, emotional, and mental consequences that might have been avoided had their depression been attended to.

It is necessary for the medical community to continue to explore the topic of how to treat this specific patient population. It was very difficult to find any studies or guidelines on how to approach this patient’s condition. Instead, many of the studies in the literature were focused on management of depression after ischemic strokes. There also needs to be more studies on the effects of SSRIs on ICH expansion/recurrence. Additionally, it is worth noting that some of the studies referenced in this case report used small sample sizes from one hospital or clinical location, which could compromise the generalizability of the study’s results.
